# The associations between the FAGR and all-cause and cardiovascular mortality in patients with STEMI

**DOI:** 10.1038/s41598-025-93951-0

**Published:** 2025-03-18

**Authors:** Sirui Yang, Yanji Zhou, Dan Xu, Yilong Dong, Haoran Tang, Pan Jing, Ya-nan Lu, Minjing Yuan, Zhensong Zhao, Lixing Chen

**Affiliations:** 1 Department of Cardiology, Kunming Medical University First Affiliated Hospital, Kunming, Yunnan Province China; 2 Department of Pediatrics, Kunming Medical University First Affiliated Hospital, Kunming, Yunnan Province China; 3 Curtin Medical School, School of Public Health, Faculty of Health Sciences, Curtin University, Perth, Australia; 4 School of Medicine, Yunnan University, Kunming, Yunnan Province China; 5 Kunming Medical University First Affiliated Hospital, The First Clinical College of Kunming Medical University, Kunming City, Yunnan Province China

**Keywords:** Fibrinogen-to-albumin-to-globulin ratio, Mortality, Prognosis, Myocardial infarction, Cardiology, Medical research, Epidemiology, Cardiovascular diseases

## Abstract

Although the fibrinogen-to-albumin-to-globulin ratio (FAGR) has been proven to be related to coronary artery disease (CAD), the association between the FAGR and acute ST-segment elevation myocardial infarction (STEMI) has not been adequately investigated. This study aimed to evaluate the prognostic potential of the FAGR for STEMI. A total of 1042 patients with STEMI after emergency PCI admitted to the First Affiliated Hospital of Kunming Medical University from June 2018 to January 2023 were enrolled in the study. Patients were divided into a low FAGR group and a high FAGR group according to the median FAGR (2.44). We used Kaplan–Meier plots, restricted cubic spline regression, Cox survival analyses and time-dependent ROC analyses to explore the predictive value of the FAGR for all-cause and cardiovascular mortality. Kaplan‒Meier analysis revealed that the cumulative incidence rates of all-cause and cardiovascular mortality in patients with STEMI were greater in the high FAGR group. Multivariate Cox proportional hazard analysis revealed that the FAGR was an independent predictor of both all-cause and cardiovascular death. In terms of the prediction of all-cause mortality, the FAGR had an area under the ROC curve of 0.720, which was better than that for fibrinogen (AUC = 0.687). In terms of the prediction of cardiovascular mortality, the area under the ROC curve for the FAGR was 0.726, which was also better than that for Fib (AUC = 0.698). The present results suggest that the FAGR may serve as a potential prognostic indicator in patients with STEMI after emergency PCI.

## Introduction

Coronary atherosclerotic heart disease is the leading cause of morbidity and mortality worldwide. Acute ST-segment elevation myocardial infarction (STEMI) is the most serious type of coronary heart disease (CHD) and has the characteristics of acute onset, rapid progression and high mortality. Although substantial progress has been made in drug therapy and myocardial reperfusion^1^, the mortality rate is still high^2,3^. Therefore, efforts are still needed to find more effective predictors to improve the prognosis of STEMI-related mortality.

Atherosclerosis (AS) is considered a chronic inflammatory disease and the most important pathological basis of coronary heart disease (CHD). It has been proposed that endothelial dysfunction is the first step in atherosclerosis^4^. Inflammatory factors play a key role in the early steps of atherogenesis in lesion-prone areas. STEMI pathophysiology involves coronary plaque rupture, plaque erosion, progressive stenosis due to plaque progression and coronary artery spasm^5^. Approximately 64% of acute coronary syndrome (ACS) cases are caused by inflammation-induced rupture of a lipid plaque with the subsequent formation of a platelet-rich thrombus, and STEMI occurs when the thrombus completely occludes the vessel^6^. Plaque erosion (thrombus formation in the fibrous intima‒media plaques rather than in the necrotic cores of the plaques) is the diametrical opposite of plaque rupture. The myocardial injury caused by acute STEMI initiates a systemic inflammatory response^7,8^. Many cells and molecules are released into the circulation, making them potential biomarkers that could be used to stratify risk among STEMI patients^9^.

Albumin (Alb) and globulin (Glo) are the main components of serum proteins. Albumin is the most abundant protein in plasma and has multiple functions: it is involved in colloid osmotic pressure formation, transporter function, acts as an antioxidant and maintains microvascular integrity^10^. Albumin is a negative acute phase reactant and is reduced in acute and chronic inflammatory states. Globulin co-exists with acute phase reactants. Thus, high levels of globulin are indicative of increased inflammatory processes and cytokine production^11^. Globulin is the main executor of immune function, and the immune system is closely connected with the development of atherosclerosis^12^. Low albumin and high globulin levels are associated with cardiovascular events^13,14^. The albumin to globulin ratio ( AGR ) has been used as an estimate of plasma viscosity. Low serum albumin is associated with reduced fibrinolysis and enhanced platelet aggregation^15^. A high ratio of fibrinogen (Fib) to albumin accelerates the aggregation of erythrocytes in capillaries, which contributes to the development of thrombotic disorders^16^. Fibrinogen plays a role in platelet aggregation, plasma viscosity, and fibrin formation^17^. Fibrinogen has been widely used in the study of coronary heart disease because of its involvement in coagulation, a major component of thrombosis. Fibrinogen is also strongly associated with inflammation and may regulate the inflammatory response by inducing the production of proinflammatory cytokines by peripheral blood mononuclear cells.

Many clinical laboratory indices have been shown to be associated with the risk of mortality in patients with STEMI and can be used to assess prognosis. Some studies have investigated the effects of the serum fibrinogen concentration and AGR on the severity and prognosis of cardiovascular disease, but the effects of the fibrinogen-to-albumin-to-globulin ratio (FAGR) on all-cause and cardiovascular mortality in patients with STEMI have not been investigated.

## Materials and methods

### Study population

A single-centre retrospective analysis of 1341 consecutive patients with STEMI admitted to the First Affiliated Hospital of Kunming Medical University from June 2018 to November 2023 was carried out. STEMI was defined according to the 2023 ESC Guidelines for Management of Acute Coronary Syndromes^18^. The enrolled patients with STEMI were undergoing emergency percutaneous coronary intervention (PCI). Patients were excluded from the study if they met the following criteria: missing necessary data (e.g., routine blood test results or cardiac ultrasound data), Lack of follow-up data (loss of contact or refusal to co-operate with follow-up at the time of telephone follow-up), or comorbidity with other serious diseases (e.g. history of autoimmune diseases, systemic diseases, malignant neoplasms, acute infections, and severe hepatic or renal insufficiency: Child classification III; creatinine clearance ≤ 20%). Finally, 1042 patients were enrolled in the study.

### Data collection and definitions

Demographic and clinical data were collected at admission. Demographics, including age, sex, history of hypertension and diabetes, family history of cardiovascular disease, and smoking and drinking status, were recorded. All laboratory data, including routine blood tests (white blood cell (WBC), red blood cell (RBC), platelet (PLT), neutrophil, monocyte, etc.), fibrinolytic function (prothrombin time, activated partial thromboplastin time, international normalized ratio (INR), and D-dimer), sodium, potassium, chlorine, biochemical indicators (total protein (TP), albumin (Alb), globulin (Glo), prealbumin, creatinine (Cre), urea nitrogen, alanine aminotransferase (ALT), aspartate transaminase (AST), glucose, cholesterol, triglyceride (TG), etc.), electrocardiograms and cardiac ultrasound data, were collected at the first visit. We also recorded the angiographic results for the patients obtained in the cardiac catheterisation operating room of the First Affiliated Hospital of Kunming Medical University, including the extent of the lesion, lesion site, degree of lesion stenosis, stent implantation site and number, and preprocedure TIMI grade.

The researchers collected survival data via telephone interviews with patients or their families. Follow-up ended at the time of the patient’s last available medical record if no response was received. All-cause mortality and cardiovascular mortality were the primary endpoints of the study.

The FAGR was defined as the ratio of concentration of fibrinogen (g/L) to the AGR. The value of the AGR was calculated by dividing the albumin concentration (g/L) by the globulin concentration (g/L).

### Statistical analysis

We divided the patients into a low FAGR group (FAGR < 2.44, *n* = 513) and a high FAGR group (FAGR ≥ 2.44, *n* = 529) according to the median FAGR. Descriptive statistics are presented as the means ± standard deviations (SDs) for normally distributed continuous variables or the medians (interquartile ranges [IQRs]) for nonnormally distributed data and as frequencies (percentages) for categorical variables. The independent sample t test was used for normally distributed numerical variables, and the Mann‒Whitney U test was used for numerical variables that were not normally distributed or exhibited heteroscedasticity. The χ^2^ test was used to compare categorical variables between groups. Differences were considered statistically significant at *P* < 0.05.

The Kaplan‒Meier method was used to estimate the probabilities of all-cause mortality and cardiovascular mortality, and the log-rank test was used to evaluate discrepancies in the curves. Univariate Cox proportional risk regression analyses were used to determine the effects of each variable on all-cause and cardiovascular mortality. Multivariate analyses with a Cox proportional hazards regression model for all-cause and cardiovascular mortality were performed using the factors found to be significant in univariate analysis. The risk ratios (HRs) were calculated, and the results are reported as HRs and 95% confidence intervals (CIs). Receiver operating characteristic (ROC) curves were plotted to determine the diagnostic value of the FAGR for STEMI prognosis, and the area under the curve (AUC) and 95% confidence interval (CI) were calculated and compared among different biomarkers. The subgroup analyses were performed via stratified Cox proportional hazards models.

## Results

### Baseline patient characteristics

In this study, we included 1042 patients (age: 60.5 ± 12.15 years) with STEMI who underwent emergency PCI. Over a median follow-up of 1090 days, 120 patients (11.52%) died from all causes, and 107 patients (10.27%) died due to cardiovascular causes. On the basis of the median FAGR, we divided the patients into two groups: the low FAGR group (FAGR-L), with a FAGR < 2.44, and the high FAGR group (FAGR-H), with a FAGR ≥ 2.44. Compared with those in the FAGR-L group, patients in the FAGR-H group had a greater average age; faster heart rate; higher PLT, Fib, Cre and UA levels; higher Gensini score and GRACE score; a greater proportion of Killip class > 1 and diseased vessels; a greater occurrence of hypertension, diabetes, hypertension and heart failure; and relatively lower RBC, Hb, Alb and ALT levels (*p* < 0.05) (Table 1).

**Table 1:** Baseline characteristics according to the FAGR.

Characteristics	FAGRL (*n* = 513)	FAGRH (*n* = 529)	*p* Value
Basic characteristics			
Sex, male, n (%)	457(89.1)	424(80.2)	< 0.001
Age (years)	58.29 ± 11.81	62.74 ± 12.08	< 0.001
HR (beat/minute)	75.70 ± 16.22	81.65 ± 17.73	< 0.001
Systolic BP (mmHg)	127.09 ± 22.95	126.1 ± 24.86	0.505
Diastolic BP (mmHg)	82.10 ± 15.84	80.61 ± 16.79	0.143
BMI (kg/m^2^)	24.31 ± 3.00	24.20 ± 3.36	0.567
Killip class > 2, n (%)	148(28.8)	204(38.6)	< 0.001
Smoker, n (%)	306(59.6)	387(73.2)	0.079
Alcohol consumption, n (%)	98(19.1)	109(20.6)	0.544
Angiographic and procedural			
Culprit artery, n (%)			
RCA	230(44.8)	225(42.5)	0.618
LM	5(1.0)	6(1.1)	0.881
LAD	260(50.7)	313(59.2)	0.415
LCX	74(14.4)	85(16.1)	0.119
LAD	260(50.7)	313(59.2)	0.415
LCX	74(14.4)	85(16.1)	0.119
Average length of implanted stents	24.02 + 6.50	24.80 + 6.50	0.795
Average diameter of implanted stents	3.00 (2.75, 3.50)	3.15 (2.75, 3.50)	0.153
Preprocedure TIMI III grade, n (%)	501(97.7)	517(97.7)	0.875
Medical history			
Hypertension, n (%)	260(50.7)	325(61.4)	< 0.001
Diabetes, n (%)	141(27.5)	186(35.2)	0.008
Hyperlipidaemia, n (%)	191(37.2)	244(46.1)	0.001
Heart failure, n (%)	105(20.5)	181(34.2)	< 0.001
Laboratory indicators			
cTnI (ng/L)	1.52(0.1,12.348)	2.57(0.16,18.66)	0.013
WBC (10^9/L)	10.97(8.4, 12.72)	11.26(8.12, 13.09)	0.863
RBC (10^12/L)	5.03 ± 0.63	4.80 ± 0.71	< 0.001
Hb (g/L)	156.63 ± 19.10	149.3 ± 26.37	< 0.001
PLT (10^9/L)	214.45 ± 66.00	232.91 ± 75.19	< 0.001
Fib (g/L)	2.57(2.33, 2.96)	4.29(3.38, 4.76)	< 0.001
Alb (g/dL)	41.13 ± 4.29	38.20 ± 4.65	< 0.001
Glucose (mmol/L)	7.40(5.09, 8.31)	7.69(5.04, 8.94)	0.296
ALT (IU/L)	58.63(30.93, 63.00)	55.68(28.00, 63.00)	0.021
AST (IU/L)	154.76(31.00, 193.65)	133.40(31.90, 166.00)	0.301
Cre (µmol/L)	92.59(76.05, 100.4)	103.88(78.00, 108.70)	< 0.001
UA (µmol/L)	386.82(315.70, 459.85)	415.79(322.70, 483.40)	0.011
TC (mmol/L)	4.48 ± 1.16	4.46 ± 1.19	0.819
TG (mmol/L)	1.85(1.03, 2.08)	1.77(1.05, 2.03)	0.735
HDL-C (mmol/L)	1.09(0.88, 1.21)	1.51(0.88, 1.22)	0.766
LDL-C (mmol/L)	2.89(2.16, 3.45)	2.99(2.21, 3.52)	0.775
Medication use, n (%)			
ACEI/ARB/ARNI	221(43.1)	243(46)	0.045
Aspirin	500(97.5)	523(98.9)	0.051
P2Y12-blocker	489(95.3)	512(96.7)	0.032
Beta-blocker	365(71.3)	402(76.0)	0.023
Statins	498(97.1)	505(95.4)	0.087
LVEF (%)	61.98 ± 10.90	60.92 ± 12.22	0.150
Gensini score	66.88(40.00, 86.00)	74.61(45.00, 96.00)	< 0.001
GRACE score	144.92 ± 33.43	158.48 ± 34.59	< 0.001

Differences in normally distributed continuous variables were compared via variance analyses, and those in nonnormally distributed data were compared via Mann‒Whitney U tests. Chi-square tests were used to compare differences in categorical variables between groups. P values were derived from comparisons of the FAGRH group and the FAGRL group. *P* < 0.05 was considered indicative of statistical significance.

Number of stents implanted: includes drug balloons with stents.

*cTnI* cardiactroponin I,* HR* heart rate,* BP* blood pressure,* BMI* body mass index,* WBC* white blood cells, *RBC* red blood cells,* NBC* neutrophil,* LBC* lymphocyte,* Hb* haemoglobin,* PLT* platelet, *Fib* fibrinogen,* Alb* albumin,* ALT* alanine aminotransferase,* AST* aspartate aminotransferase,* Cre* creatinine,* UA* uric acid,* GFR* glomerular filtration rate,* TC* total cholesterol, *TG* triglyceride,* HDL-C* high-density lipoprotein cholesterol,* LDL-C* low-density lipoprotein cholesterol, *ACEI* angiotensin converting enzyme inhibitor, *ARB* angiotensin II receptor blocker, *ARNI* angiotensin receptor-neprilysin inhibitor, *LVEF* left ventricular ejection fraction.

### Associations between FAGR and all‑cause and cardiovascular mortality

**Fig. 1:**
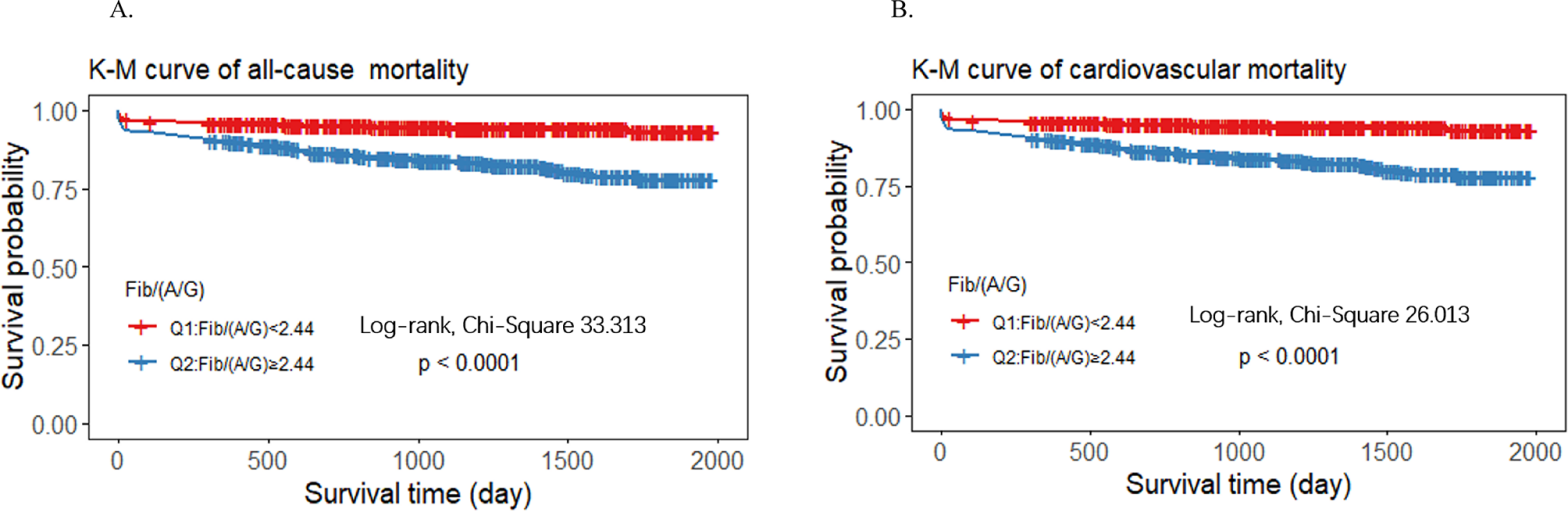
Kaplan‑Meier survival curves (**A**). all-cause mortality, (**B**). cardiovascular mortality on the basis of FAGR levels

We performed Kaplan‒Meier curve analysis on the basis of the median FAGR to explore the relationships between the FAGR and all-cause and cardiovascular mortality. The results shown in Fig. 1 indicate that the risks of all‑cause mortality (log-rank χ 33.313 *p* < 0.001) and cardiovascular mortality (log-rank χ 26.013 *p* < 0.001) were significantly greater in patients with higher FAGR values.

**Fig. 2:**
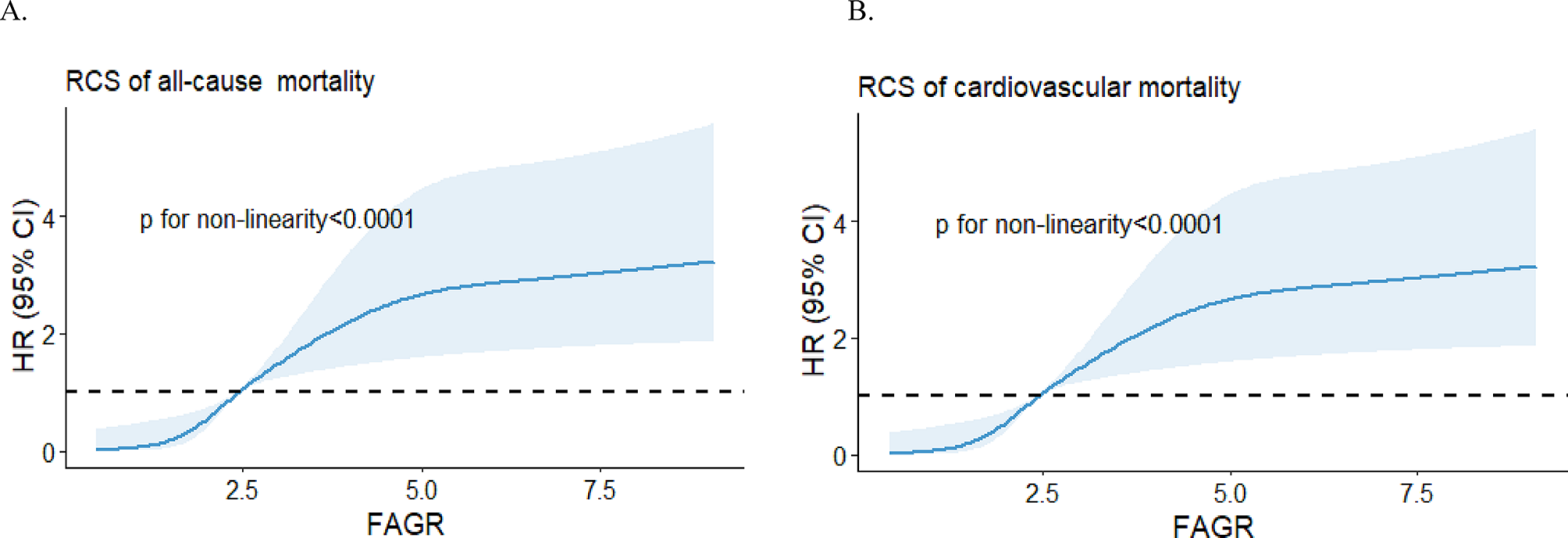
Restricted cubic spline analysis for the associations between the FAGR and (**A**). all-cause mortality, (**B**). cardiovascular mortality.Note: Hazard ratios are indicated by solid lines, and 95% CIs are indicated by shaded areas.

As shown in Fig. 2, we used restricted cubic splines to flexibly model and visualize the relationships of the FAGR with all-cause and cardiovascular mortality in STEMI patients after emergency PCI. The FAGR was broadly positively associated with both all-cause and cardiovascular mortality in patients with STEMI. The trend of an increasing risk of mortality with increasing FAGR plateaued after the FAGR was greater than approximately 5.

### FAGR as predictor of adverse outcomes

To further determine the role of the FAGR in mortality, we performed univariate and multivariate Cox proportional hazard model analyses. (Table 2.) Univariate Cox proportional hazard analysis revealed that FAGR was a risk factor for all‑cause mortality (HR: 3.245, 95% CI: 2.124–4.956, *p* < 0.001) and was an independent risk factor for cardiovascular mortality (HR: 2.998, 95% CI: 1.923–4.673, *p* < 0.001). After adjustment for age, sex, BMI, smoking status, hypertension status, diabetes status, hyperlipoidaemia, RBC, Hb, Cre, left main or multivessel disease and GRACE scores, multivariate Cox proportional hazards analysis suggested that the FAGR was still independently correlated with all-cause mortality (HR: 1.896, 95% CI: 1.185–3.304, *p* = 0.008) and cardiovascular mortality (HR: 1.684, 95% CI: 1.027–2.763, *p* = 0.039).

**Table 2:** Univariable and multivariable analysis with Cox proportional hazards models.

	Univariable		Multivariable	
	HR (95% CI)	*p*	HR (95% CI)	*p*
Allcause mortality				
FAGR	3.245(2.124, 4.956)	< 0.001	1.896(1.185, 3.034)	0.006
Age	1.083(1.065, 1.102)	< 0.001	1.030(1.008, 1.054)	0.009
Female (reference: Male)	2.234(1.489, 3.352)	< 0.001		
BMI	0.947(0.890, 1.006)	0.075		
Smoking	1.962(1.363, 2.825)	< 0.001		
Hypertension	2.098(1.403, 3.316)	< 0.001	1.476(1.012, 2.300)	0.048
Diabetes	1.626(1.128, 2.342)	0.009		
Hyperlipoidemia	2.270(1.403, 3.672)	0.001	1.865(1.084, 3.210)	0.024
RBC	0.650(0.516, 0.818)	< 0.001		
Hb	0.985(0.978, 0.992)	< 0.001		
Cre	1.003(1.001, 1.004)	< 0.001		
GRF	0.966(0.959, 0.974)	< 0.001		
Left main or multivessel disease	2.567(1.253, 5.261)	0.01		
GRACE score	1.026(10.22, 1.030)	< 0.001	1.020(1.014, 1.026)	< 0.001
Cardiovascular mortality				
FAGR	2.998(1.923, 4.673)	< 0.001	1.701(1.051, 2.754)	0.031
Age	1.080(1.060, 1.099)	< 0.001		
Female (reference: Male)	2.428(1.588, 3.713)	0.001		
BMI	0.954(0.895, 1.017)	0.895		
Smoking	1.993(1.348, 2.945)	0.001		
Hypertension	2.402(1.540, 3.748)	< 0.001	1.722(1.053, 2.816)	0.023
Diabetes	1.756(1.191, 2.588)	0.004	1.528(1.004, 2.325)	0.045
Hyperlipoidemia	2.082(1.265, 3.424)	0.004	1.806(1.026, 3.179)	0.04
RBC	0.639(0.500, 0.815)	< 0.001		
Hb	0.984(0.977, 0992)	< 0.001		
Cre	1.003(1.001, 1.004)	0.001		
GRF	0.967(0.959, 0.974)	< 0.001		
Left main or multivessel disease	3.660(1.490, 8.988)	0.005	3.001(1.086, 8.295)	0.032
GRACE score	1.026(1.022, 1.031)	< 0.001	1.021(1.015, 1.028)	< 0.001

Ref.: FAGR-L group (FAGR < 2.44).

*FAGR*: fibrinogen-to-albumin-to-globulin ratio,* BMI* body mass index,* RBC* red blood cells,* Hb* haemoglobin,* Cre* creatinine,* Cre* creatinine,* GFR* glomerular filtration rate,* HR* hazard ratio,* CI* confidence interval.

Table 3 shows the four multivariate Cox proportional hazard models used to determine the correlations between the FAGR groups and outcomes. In Model 4, the covariates adjusted for were age, sex, BMI, RBC, Hb, Cre, GRF, sodium, potassium, chloride, hypertension, diabetes, hyperlipoidaemia, left main or multivessel disease and GRACE score. With the low FAGR group used as a reference, a high FAGR was associated with higher incidences of all-cause mortality and cardiovascular mortality.

**Table 3:** Cox proportional hazards models for the associations between FAGR and mortality.

	Allcause mortality		Cardiovascular mortality	
	HR (95% CI)	p	HR (95% CI)	p
Unadjusted	3.245(2.124, 4.956)	< 0.001	2.998(1.923, 4.673)	< 0.001
Model 1	2.222(1.440, 3.430)	< 0.001	2.072(1.315, 3.265)	0.002
Model 2	2.036(1.306, 3.172)	0.002	1.902(1.195, 3.030)	0.007
Model 3	1.852(1.183, 2.900)	0.007	1.700(1.062, 2.721)	0.027
Model 4	1.864(1.181, 2.944)	0.008	1.690(1.045, 2.734)	0.032

Ref.: FAGR-L group (FAGR < 2.44).

Model 1: Adjusted for age, sex, and BMI;

Model 2: Adjusted for M1 + RBC, Hb, Cre, and GRF;

Model 3: Adjusted for M2 + hypertension, diabetes, and hyperlipidaemia;

Model 4: Adjusted for M3 + left main or multivessel disease and the GRACE score.

### Predictive ability of the FAGR in patients with STEMI

**Fig. 3:**
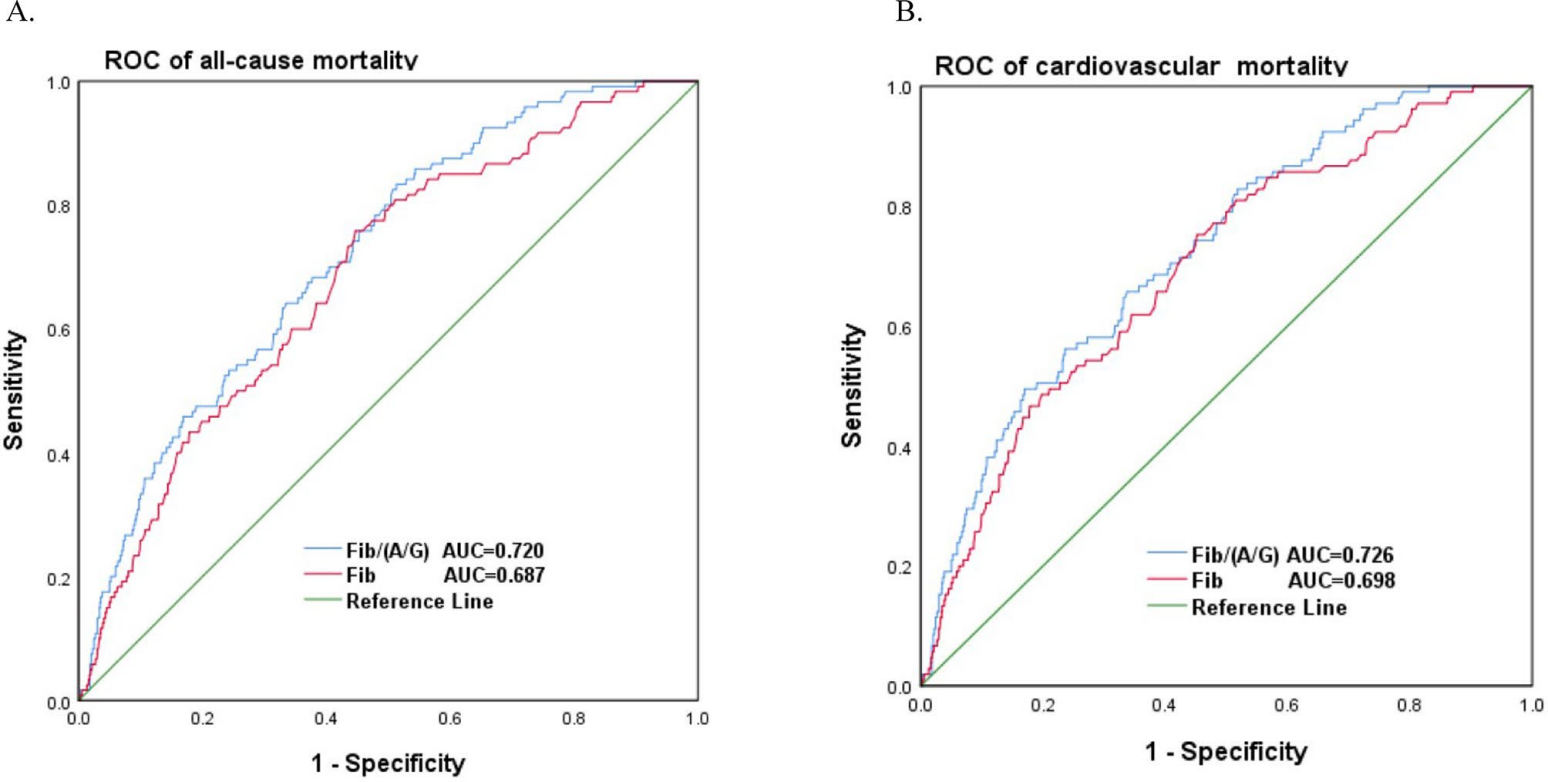
ROC curves of the FAGR with the reference line for (**A**). all-cause mortality, (**B**). cardiovascular mortality.

We generated time‒dependent ROC curves to evaluate the prognostic value of the FAGR for all-cause mortality and cardiovascular mortality. (Fig. 3) According to the ROC curve analysis, the FAGR had an AUC of 0.720 (95% CI 0.675–0.765), with a sensitivity of 82.5% and a specificity of 49.2%, for predicting all‑cause mortality. FAGR was significantly better than Fib (AUC = 0.687, 95% CI 0.639–0.735, *P* < 0.001). With regard to predicting cardiovascular mortality, the FAGR had an AUC of 0.726 (95% CI 0.678–0.774), with a sensitivity of 56.2% and a specificity of 76.3%, which was also better than the results for Fib (AUC = 0.698, 95% CI 0.646–0.749).

## Discussion

This retrospective study was conducted to demonstrate that the FAGR has a significant effect on prognosis in patients with STEMI. This is the first study reporting the prognostic value of the FAGR in STEMI patients. All-cause mortality and cardiovascular mortality were higher in the high FAGR group than in the low FAGR group. Meanwhile, FAGR was an independent predictor of all-cause and cardiovascular mortality in STEMI patients, and FAGR was better than Fib in predicting either all-cause or cardiovascular mortality.

Atherosclerosis is recognized as a chronic inflammatory disease and is the most important pathological basis of coronary heart disease. Coronary plaque rupture, plaque erosion, progressive stenosis due to plaque progression, and coronary artery spasm all contribute to the development of STEMI. STEMI also occurs when inflammation-induced rupture of a lipid plaque induces the formation of a platelet-rich thrombus, which completely occludes the vessel.

As the most abundant plasma protein component, albumin protects the vascular wall by playing an antioxidant role against oxidative damage to the vascular endothelium, therefore inhibiting the progression of atherosclerosis. Studies have shown that serum albumin levels are negatively correlated with cardiovascular outcomes. Albumin is involved in acute and chronic inflammatory reactions and is negatively correlated with the degree of the inflammatory reaction^19^. In addition, serum albumin is an important inhibitor of platelet activation and aggregation and is an important mediator of platelet-induced coronary artery constriction^20^. Chronic inflammatory processes and abnormal immune function may also be involved in atherosclerosis development. Globulin is the main executor of immune function, and the immune system is closely connected with the development of atherosclerosis^12^. Research has suggested that globulin contains antiatherosclerotic components (e.g., γ-globulin)^21^. Globulin coexists with acute-phase reactants (such as fibrinogen, complement factor and C-reactive protein), and the level of globulin increases due to the inflammatory process and increased cytokine production. High immunoglobulin levels are especially apparent in patients with coronary heart disease or acute coronary syndrome. In addition to its role as an inflammatory marker, globulin mediates the progression of atherosclerosis by subendothelial spreading with lipid particles, lymphocytes and macrophages^22^. The albumin-to-globulin ratio (AGR) is obtained by dividing the concentration of albumin by that of globulin, a recognized marker of inflammation that has been widely used to assess the prognosis of cancer^23–25^. In the nervous system, the AGR is considered an important factor protecting against cognitive decline^26^. In addition, in circulatory diseases, a lower AGR is closely related to the occurrence of malignant cardiovascular and cerebrovascular events30 and mortality in patients with heart failure with a reduced ejection fraction^27,28^. A few studies have shown that the levels of both serum albumin and globulin are associated with CHD, independent of traditional CHD risk factors^29^.

Fibrinogen is widely used in the study of coronary artery disease because fibrinogen is involved in the coagulation process and is a major component of thrombosis. Fibrinogen plays a role in platelet aggregation, plasma viscosity, and fibrin formation and is an indicator of coagulation status^16^. Relevant studies have confirmed that a high level of plasma fibrin promotes a change in the permeability of the atherosclerotic macrophage cap, which leads to a series of changes, such as the thinning of the plaque cap. At that point, the arterial plaque will rupture, leading to the formation of a thrombus^30^. Fibrinogen, an acute time-phase protein, is closely associated with inflammation and may be involved in thrombosis by inducing the production of proinflammatory factors by peripheral blood monocytes to produce the proinflammatory cytokines interleukin-1 (IL-1), interleukin-6 (IL-6) and tumour necrosis factor-α (TNF-α) to regulate the inflammatory response. Chronic inflammation due to atherosclerosis further contributes to elevated plasma fibrinogen levels^31^.

Fibrinogen and globulin are positively correlated with the inflammatory response, whereas albumin is negatively correlated with the inflammatory response, and the interaction of albumin with fibrinogen can lead to impaired fibrinogen activity^32^. Zhen Wang et al. demonstrated that the FAGR is a valuable potential new inflammatory marker^33^, and Bülent Deveci et al. reported that the FAGR is strongly associated with the presence and severity of coronary artery disease and can be used to predict cardiovascular risk^34^. However, no study has explored the relationship between the FAGR and prognosis in STEMI patients. Having clarified the effect of the FAGR on all-cause and cardiovascular deaths in STEMI patients, we suggest that the FAGR may be a valuable composite biomarker for the prediction of the risk of cardiovascular events, the identification of patients at a high risk for STEMI, and the guidance of follow-up after individualized treatment for STEMI patients.

## Conclusion

This study indicates that the FAGR is an independent predictor of prognosis in STEMI patients who underwent emergency PCI.

Our study demonstrated that the all-cause and cardiovascular mortality rates in the high FAGR group were greater than those in the low FAGR group.

### Limitations

This was a single-centre study with limited clinical data. This was a retrospective observational study, and data bias could not be avoided although there was correction for multiple confounding factors. Further prospective studies are needed to validate the role of the FAGR in the prognosis of STEMI.

## Data Availability

The datasets used and/or analysed during the current study available from the corresponding author on reasonable request.
